# Establishing baseline biodiversity data prior to hydroelectric dam construction to monitoring impacts to bats in the Brazilian Amazon

**DOI:** 10.1371/journal.pone.0183036

**Published:** 2017-09-08

**Authors:** Paulo Estefano D. Bobrowiec, Valéria da Cunha Tavares

**Affiliations:** 1 Coordenação de Biodiversidade, Instituto Nacional de Pesquisas da Amazônia (INPA), Manaus, Amazonas, Brazil; 2 Departamento de Zoologia, Instituto de Ciências Biológicas, Universidade Federal de Minas Gerais (UFMG), Belo Horizonte, Minas Gerais, Brazil; 3 Programa de Pós-graduação em Genética, Conservação e Biologia Evolutiva, Instituto Nacional de Pesquisas da Amazônia (INPA), Manaus, Amazonas, Brazil; Pacific Northwest National Laboratory, UNITED STATES

## Abstract

The modification of Amazonian rivers by the construction of megaprojects of hydroelectric dams has widely increased over the last decade. Robust monitoring programs have been rarely conducted prior to the establishment of dams to measure to what extent the fauna, and its associated habitats may be affected by upcoming impacts. Using bats as models, we performed analyses throughout the area under the influence of the Santo Antônio hydroelectric dam, Southwestern Brazilian Amazonia before its construction to estimate how the fauna and its associated habitats would be affected by the upcoming impacts. We surveyed bats in 49 plots distributed along the areas going to be inundated by the dam and those remaining dry. As predictors for the species distribution, we tested the variables of vegetation structure and topography. Species composition largely differed between the dry plots and the plots located in areas that will be flooded, and this was strongly associated with the variables of forest basal area and elevation. Vegetation-related variables also had strong influence on the guilds distribution. The flooding of lower elevations areas is expected to negatively affect the species number and abundance of frugivorous species. In contrast, it is likely that animalivores will be less vulnerable to dam-induced flooding, since they were abundant in the areas not expect to be inundated. We urge for the implementation of studies to predict impacts caused by large hydroelectric dams, including tests of the influence of the local conditions that shape diversity to avoid massive losses of the biota, and to build preventive monitoring and management actions.

## Introduction

Brazilian industrial and economic development coupled with demographic expansion, contributed to >40% increase in energetic demands within a single decade, beginning in 2001 [1]. Approximately 65% of the energy currently generated for the Brazilian national grid comes from the hydroelectric power stations, which have been promoted as cleaner and less expensive sources of energy by most governmental agencies [2,3]. However, several studies have shown unequivocally that hydroelectric energy production imposes severe impacts on the regional biota [4–6]. Though many are long-term, they begin with those caused by the temporary or permanent flooding of the area of the dam and its surroundings. The construction of hydroelectric stations indeed triggers significant environmental and social impacts, but historically in Brazil, studies have underestimated the impacts of hydroelectric dams and overestimated their benefits [7,8].

Recently, Lees et al. [9] declared that the “Amazon has become synonymous with dam development”. In fact, the plan of energetic expansion 2017–2021 from the Ministry of Mines and Energy of Brazil [2] indicates that nine large hydroelectric power stations, totaling to over 30 MW of capacity, will be built on rivers in the Brazilian Amazonia. Over 80 additional hydroelectric power stations will be constructed in other neighboring Amazonian countries [10].

Modifications to natural landscapes by hydroelectric power stations interfere with the distribution and abundance of species by altering the quality of habitats [5,6,11]. It is known that different species do not use the same habitats in the same way, as they may concentrate in specific areas within ecological gradients (e.g. for frogs [12]; for large mammals [13]; for bats [14]). Anthropogenic alteration of such gradients causes direct disturbance to relative abundance of the species, and to species composition, in particular those species directly associated to the affected gradients.

Variation in vegetation, topography, and soil properties have been considered important predictors of the structure of animal assemblages [15,16]. Topography may produce gradients of water availability [17], texture and fertility of the soil [18], light permeability through the habitats, and canopy openness [19]. Topographical features have been considered the most determinant factors affecting the species distribution of the understory shrubs and trees in the Amazonia [18,20,21]. Plant communities, in turn, shape the architecture of the forests, influence in the microclimate regulation, and in the production of feeding resources to a variety of consumers, which may become specialized and occupy different niches [22,23].

Bats are excellent indicators of the health of ecosystems, and an outstanding model group with which to evaluate anthropic effects on natural communities, particularly the Neotropical Phyllostomidae bats, which are highly diverse both in species richness and in ecology [24]. Habitat loss from human interference has repeatedly been identified as the main threat to the survival of many bat species [11,25,26], but the effects of the establishment and operation of hydroelectric power stations on bat assemblages, and to the quality of the habitats they occupy, are still poorly understood.

Most studies of bat assemblages from areas under the influence of hydroelectric power stations have been conducted after the flooding of reservoirs with the goals of evaluating the effects of the insularization of the native environments [11,27–29]. In contrast, to our knowledge only a single study has offered predictions of the effects of the establishment of the hydroelectric prior to the flooding and its real consequences [27]. Here, we analyzed the bat assemblages associated with the Santo Antônio power station area (UHE Santo Antônio) located in the upper Madeira River, southern Amazonia, over different levels of organization (composition, richness and abundance of species and guilds) and habitat structure (vegetation and topography). We conducted our samplings prior to the construction of the power station comparing bat assemblages from areas to be submerged after the flooding, with “control areas”, which remained dry.

Specifically, we tested whether bat assemblages from areas remaining dry after the formation of the dam were different from the areas that will be underwater, and investigated how bat assemblages were associated with the vegetation structure and topography. We also analyzed how assemblage structure, gradients of the vegetation, and topography interact, how they will respond to the flooding of the original landscapes, and considered what would be the implications of such responses for the biological conservation of the bat fauna. As the formation of the dam was planned in a fashion that restricts submersion mostly to flat and low-lying areas, we predicted that bat assemblages sampled in plots in low areas to be submerged would differ from plots not affected by the UHE Santo Antonio reservoir. We also expected that variables associated to the vegetation and to the topography influence in the spatial distribution of the bats. If bat species composition differs between flooded and non-affected areas, terrain elevation may emerge as the strongest correlate of differences between these two areas.

## Methods

### Ethics statement

Experienced investigators handled all captured bats. We followed the guidelines approved by the American Society of Mammalogists in our procedures [30]. Systematic series of specimens were collected and all voucher specimens were deposited in the Mammal Collections of the Instituto Nacional de Pesquisas da Amazônia (INPA 6010–6276). Bats were euthanized humanely by sedation using ethyl ether followed by cervical dislocation. This study was undertaken under licenses for scientific purposes (capture, collection, and transport of specimens) from Instituto Brasileiro do Meio Ambiente e dos Recursos Naturais Renováveis—IBAMA (Procedure number 02001.000965/2008-83, Permit number 259/2009) and INPA (Memorandum of February 18, 2009).

### Study area

The Madeira River is a major tributary of the Amazon River, and has been classified as a white and muddy water river, responsible for 15% of its overall volume [31]. The average annual precipitation in the study area is 2029 mm, with a rainy period from November to April and a dry season from June to September (1998–2007 data of the National Water Agency, ANA). The vegetation of the region was originally composed by dense tropical rainforests [32], and characterized by a mosaic of vegetation types of *terra firme* forests, lowland flooded forests on the margins of the river, and patches of campinarana and campina (sand soil vegetation typical of Amazonia).

**Fig 1:**
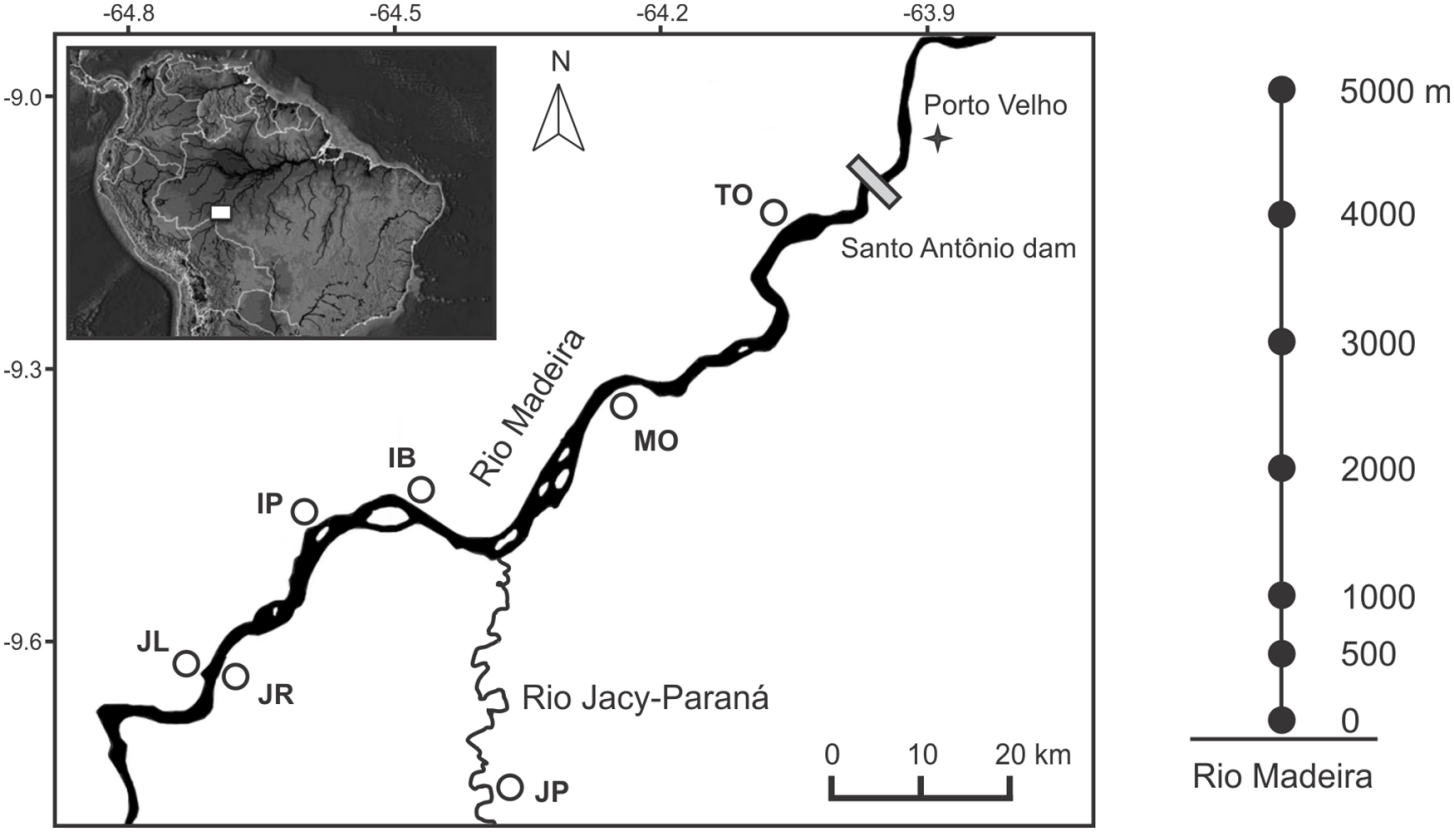
Map of the study area showing the seven sampling modules along the 100-km section of the River Madeira, as follows: TO = Teotônio, MO = Morrinhos, IB = Ilha dos Búfalos, IP = Ilha das Pedras, JL = Jirau Left Bank, JR = Jirau Right Bank, JC = Jaci. In detail (right), sampling design of each module of 5 km with seven plots (black circles) distributed 50, 500, 1000, 2000, 3000, 4000, and 5000 m from the Rio Madeira bank. All modules were arranged perpendicular to the river.

The UHE Santo Antônio is located in the upper Madeira River, close to the city of Porto Velho, Rondônia State, in the southern Brazilian Amazonia (Fig 1). UHE Santo Antônio is a large hydroelectric power station capable of generating 3150 MW and it is expected to cause the permanent flooding of 270 km^2^ of primary Amazonian rainforest surrounding the reservoir [8]. The turbines type used are Run-of-river that presumes to be less environmentally damaging than traditional dams due operate with a lower water storage using the flow within the river channel. The dam was filled in 2012. UHE Santo Antônio is located approximately 117 km west of the Jirau dam, another large hydroelectric dam with a capacity of 3750 MW [8]. The city of Porto Velho and surroundings are areas that have suffered from historical anthropogenic disturbances mainly caused by the extensive cattle ranching. The vegetation of the riverbanks within the dam's flood quota was suppressed only after the bat captures have been conducted in all sampling plots. After the dam filling, dam floodgates controlled the water level in the quota of 70 m.

### Bat captures

We captured bats with ground-level mist-nets distributed in standard sampling plots distributed through 100 km along the Madeira River margins close to the hydroelectric dam. Sampling plots were chosen to encompass gradients of vegetation and topography represented in the region. We obtained data on bat species composition with the support of the Program for Conservation of the Wildlife conducted by the Santo Antonio Energia, the company responsible for the building and operation of the Santo Antônio hydroelectric. We followed the RAPELD methods created to standardized inventories in the Amazonia [33].

Sampling design included seven sampling modules with seven plots each, totaling 49 sampling plots (Fig 1). The sampling plots were distributed at 50, 500, 1000, 2000, 3000, 4000, and 5000 m distances from the Madeira River margin in trails perpendicular to the river (Fig 1). Each plot consisted of one trail of 250 m following the contour line of the terrain, in order to minimize the topographic variations and, consequently, the variation of the vegetation inside each plot. We sampled bats in three to five nights for season (dry or rainy; S1 Appendix) between June 2010 and September 2011, totaling 217 capture nights, and 10416 mnh (1 mist-net hour, mnh, equals one 12-m net open for 1 h). To achieve this total sampling per plot we worked in three to four plots simultaneously in a same night. We gathered information on the water levels of flooding expected to be reached with the creation of the dam reservoir, and sampled 10 plots predicted to be completely submerged, and 39 predicted to remain dry (S1 Appendix).

We erected eight ground level mist-nets (12 × 3 m, 19 mm mesh, Ecotone, Poland) in each plot, arranged sequentially. The net lines started 20 m from the beginning of the centerline of each plot. The nets were left open from 18:00 h to 00:00 h, and checked at 15 minutes intervals. Bats were identified with the help of keys and descriptions found in Lim and Engstrom [34], Charles-Dominique et al. [35], and Gardner [36]. Taxonomy followed Simmons [37], with modifications found in Gardner [36] and Nogueira et al. [38]. We categorized each species within a foraging guild (frugivores, foliage-gleaning animalivores, nectarivores, omnivores, sanguinivores, and aerial insectivores), as proposed by Kalko [24]. The data and metadata of the species captured are deposited in the public repository of the PPBio (https://ppbiodata.inpa.gov.br/metacatui/#view/PPBioAmOc.87.4). The data can be accessed by title “Bat species (Chiroptera) captured in 49 sampling plots in the upper River Madeira—Rondonia, Brazil” or key words "Chiroptera", "Madeira River", and "Rondônia".

### Topography and vegetation structure in the plots

We used the variables forest basal area, vegetation clutter, elevation, and slope of the terrain to describe the structure of the landscapes in each plot. The forest basal area was calculated per plot as the total sum of DBH^2^π/4 (DBH = circumference of the tree ≥ 1 cm at the height of its breast). We measured the CBH (circumference of the tree at the height of its breast) for each tree and transformed to DBH, using the formula DBH = CBH/π. We measured trees in each 1-ha (250 × 40 m) plot, following the hierarchical size classes of CBH [39].

The vegetation density was estimated using the intercept point sampling method, which consists in quantifying the number of direct contacts by leaves and branches to a long (1.5 m in length) stick erected at 50 cm above ground, and placed perpendicular to the terrain slope [12]. We measured the number of points that touched the stick at every 2 m along the 250 m of each transect, in each plot (n = 126 touches point), and compiled the sum of contacts for each plot.

A professional surveyor using a theodolite determined the elevations of the terrains. The terrain slope was measured perpendicular to the contour line using a clinometer placed at every 50 m along the 250 m transect of the central line of each plot (n = 5 measurements). We used the mean of the five measurements as the estimate of the slope for each plot.

### Analyses

We standardized the data relative to the abundance of species and of guilds dividing raw values by the total number of individuals recorded for each plot. We included only the phyllostomid bats in our analyzes, in order to minimize bias introduced by the use of ground level mist-nets, which this close to the ground are only selectively effective at capturing representatives of Phyllostomidae bat family [24].

From the original 49 plots sampled, we excluded from analyses three with less than 10 captures, because of the potential bias they may create in the analyses and as they may have been simply collection artifacts not necessarily reflecting relative abundance of bats in these plots. Other two plots lacking data for the vegetation structure and topography and were removed from the analyses. In total we conducted analyses including 44 plots, of which 10 are to be submerged by the dam, and 34 that remained dry after the nearby flooding (S1 Appendix).

We compared the Non-metric Multidimensional Scaling ordination (NMDS) based on a Bray-Curtis similarity matrix, for species and guild composition from the sampling plots that are going to be submerged with those remaining dry. To test for differences in species and guild composition between submerged and dry sets of plots, we used an Analysis of Similarity (ANOSIM), based on Bray-Curtis similarity distances.

We employed *t*-tests of Student to compare the total richness, total abundance, and the phyllostomid guild richness and abundance between the submerged and dry sets of plots. We compare rarefied species richness between the submerged and dry sets of plots using EstimateS v. 9.1.0 [40] with 1000 randomizations. We assessed the number of species expected to occur in both areas using the nonparametric first-order Jackknife estimator. Rarefaction curves and Jackknife 1 were based on the relative abundance of the species. We estimated the expected relative abundance changes [24] related to forest flooding as the ratio (RA) between the relative abundance of species (RA = captures/mnh) in plots that going to be submerged and in those there were going to remain dry: abundance change = log [(RA_submerged plots_+0.0001)/(RA_dry plots_+0.0001)].

Possible influences of the predictor variables forest basal area, vegetation clutter, elevation, and slope were tested using Generalized Linear Mixed Models (GLMM) as implemented in the lmer function in the ‘lme4’ package [41]. The models incorporated the seven modules and plot locations (including both dry and submerse sites) as random effects to account for potential spatial autocorrelations. The response variables used for the GLMM analyses were derived from the first two ordination axes of the NMDS of the species and guild composition, the number of species, the relative abundance (bats/mnh), and the relative abundance of guilds.

We estimated the Variance Inflation Factor (VIF) to test for multicollinearity among all variables for each GLMM model, as collinearity among predictor variables may lead to incorrect identifications of predictors in multivariate models [42]. Our analysis indicated low multicollinearity (VIF < 1.7) and consequently, no predictor variable was removed from the regression models. The total variance explained by the predictor variables in a GLMM model was calculated using r.squaredGLMM function in ‘MuMIn’ package [43]. The independent contributions of each explanatory variable were estimated using hierarchical partitioning as implemented in the ‘hier.part’ package [44]. NMDS ordinations were estimated using the metaMDS function (arguments k = 2, trymax = 5000). The NMDS and ANOSIM analyzes were performed using the ‘vegan’ package [45], and the VIF analyzes were run with the help of the ‘car’ package [46]. Partial-regression plots were generated from multiple regression models using avPlots function in ‘car’ package [46], since it is not possible to generate partial plots from GLMM models. All analyses were undertaken with the R platform [47].

## Results

### Richness and relative abundance

Within the selected 44 plots (197 sampling nights, 9456 mnh), we captured 2306 bats, belonging to 58 species from six families (S2 Appendix and [48] for a complete bat inventory in the region). Species from the family Phyllostomidae represented most of the captures (n = 2229), and approximately 83% of the species recorded (n = 48). Phyllostomid richness varied from 8 to 21 species per plot (13.0 ± 2.8 species), and number of individuals per plot varied from 26 to 161 bats (50.7 ± 26.5 individuals). Most of the phyllostomid species (47.9%; 24 species) were rare (≤ 10 captures), and represented 3.6% of the total captures (84 individuals). Approximately 46% of the phyllostomid captures (22 species) were recorded in less than five plots, and 16.7% (8 species) were captured in a single plot. Nine species (18.8%) were broadly distributed across the plots, occurring in more than half of the total number of plots sampled (n = 44). No species occurred in all of the 44 sampling plots. The fruit bat *Carollia perspicillata* was the most frequently captured species, responsible for 33.2% of the total captures, and was followed by *C*. *brevicauda*, *Rhinophylla pumilio*, *Artibeus planirostris*, *A*. *obscurus*, and *A*. *lituratus*, all frugivores, that together accounted for 35% of the total of individuals captured.

### Topography and vegetation structure in the plots

The variables forest basal area, vegetation clutter, elevation, and slope varied widely among plots. The plots covered a forest basal area gradient ranging from 7.5 to 32 m^2^ (17.6 ± 4.7 m^2^; mean ± SD), while the vegetation clutter varied from 60 to 200 touches (116.1 ± 35.3 touches) across all plots. Slope varied from 0 to 15.3° (2.2 ± 2.8°), and the maximum difference of elevation among plots was 35 m, ranging from 39 to 109 m a.s.l. (84.4 ± 10.4 m). Forest basal area and vegetation clutter were variable within submerged and dry plots, and therefore no significant differences occurred between areas (*P* > 0.11). In contrast, plots that were going to be underwater had the lowest values of elevation (*t* = 10.80, *P* < 0.0001) and of slope (*t* = 2.29, *P* = 0.03).

**Fig 2:**
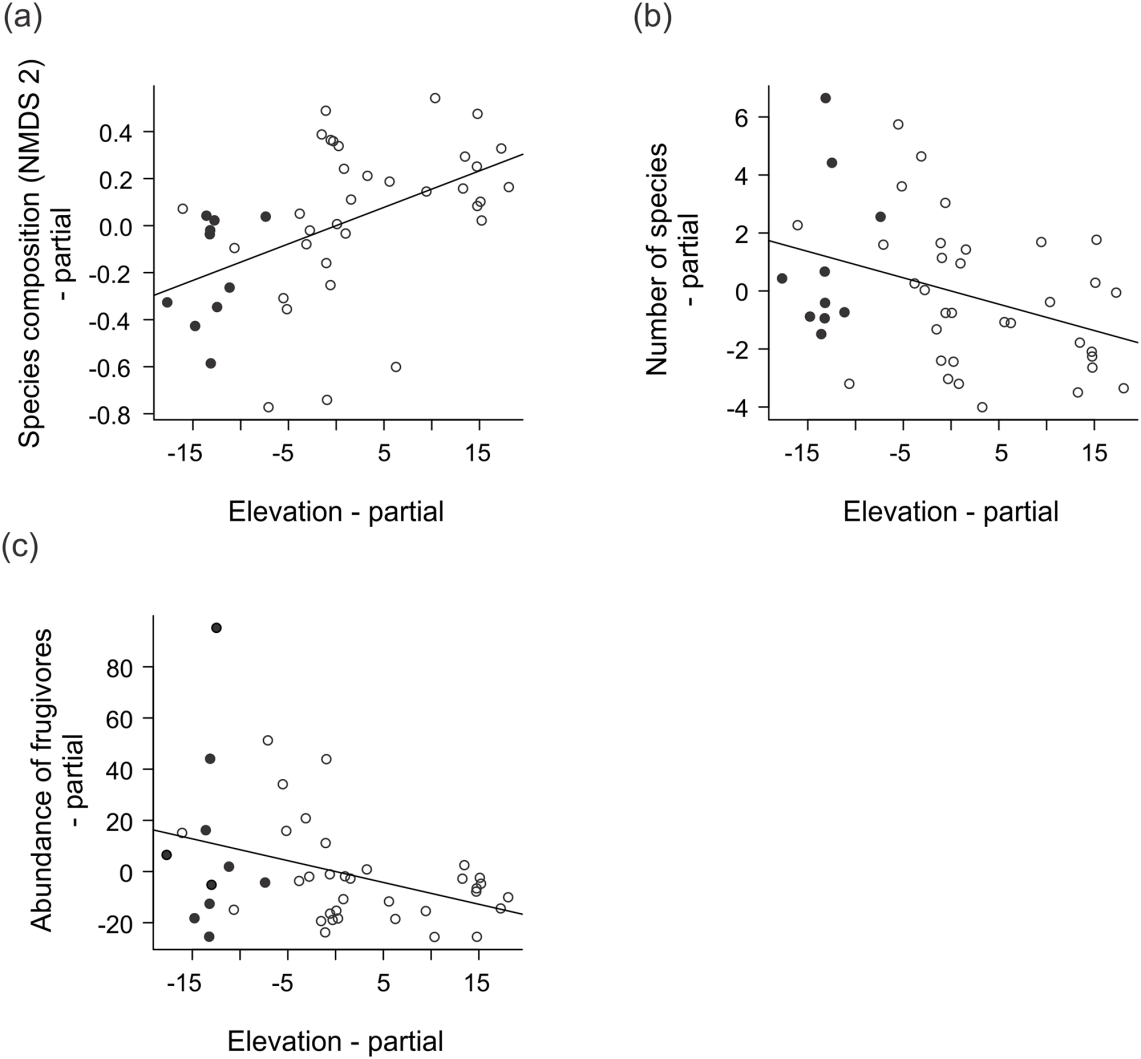
Relationships between different levels of bat community organization and topographic variables measured in 44 plots along the Madeira River, in Rondônia State, Brazilian Amazonia, between 2010 and 2011. Partial regression results between elevation and (a) species composition (NMDS axis 2), (b) number of species, and (c) abundance of frugivores. Black circles represent sampling plots that going to be submerged by the dam and white circles represent plots those that will remain dry.

**Fig 3:**
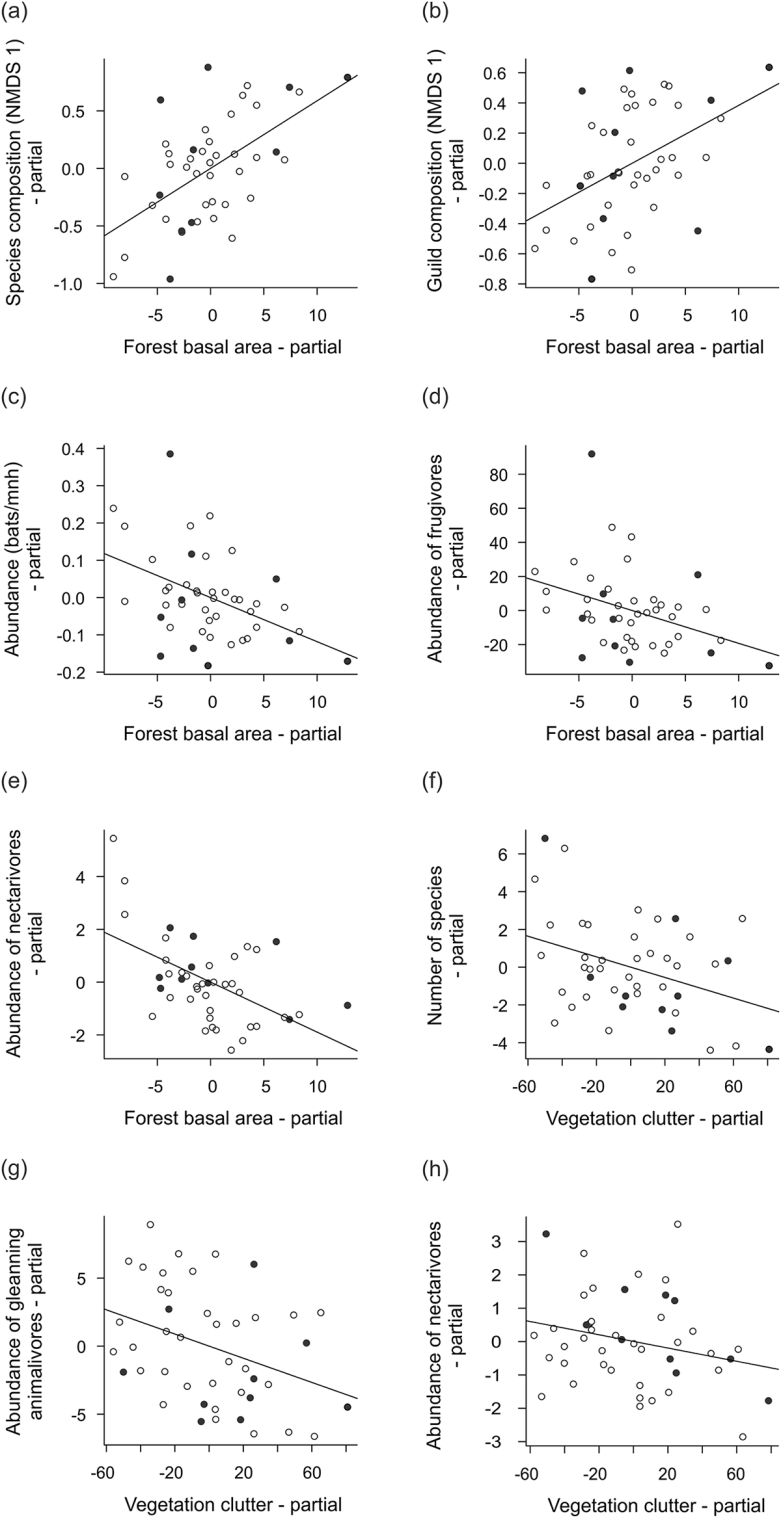
Relationships between different levels of bat community organization and variables related to the vegetation measured in 44 plots along the Madeira River, in Rondônia State, Brazilian Amazonia, between 2010 and 2011. Partial regression results between forest basal area and (a) species composition (NMDS axis 1), (b) guild composition (NMDS axis 1), (c) relative abundance (bats/mnh), (d) abundance of frugivores, (e) abundance of nectarivores, and between vegetation clutter and (f) number of species, (g) abundance of gleaning animalivores, and (h) abundance of nectarivores. Black circles represent sampling plots that going to be submerged by the dam, and white circles represent those plots that will remain dry.

The composition and relative abundance of the phyllostomid assemblages were related to the vegetation and to the topography variables (Table 1). Species and guild composition were responsive to forest basal area and elevation (Figs 2 and 3), and explained from 22% to 47% of the variation, when correlated (Table 1). Variables related to the vegetation had greater independent effects (> 38%) in the relative abundance of the species and guilds and in the number of species, when compared to those related to the topography (Table 1). The forest basal area was negatively correlated with the relative abundance of bats, and also of frugivores, and of nectarivores (Fig 3). The vegetation clutter was negatively related to the number of species and to the abundance of animalivores and of nectarivores (Fig 3). The number of bat species and the relative abundance of frugivores were negatively correlated with the elevation (Fig 2).

**Table 1:** Summary of Generalized Linear Mixed Models (GLMMs) evaluating different levels of bat community organization in relation to topographic and vegetation variables measured in 44 plots along the Madeira River, in Rondônia State, Brazilian Amazonia, between 2010 and 2011. Total explained variance of each model and independent explanatory power (HP) based on hierarchical partitioning of each significant variable are shown. Only independent power of explanatory variables with significant effect are given.

		Variance explained	Vegetation				Topography			
			Forest basal area		Vegetation clutter		Altitude		Slope	
			*t*	HP (%)	*t*	HP (%)	*t*	HP (%)	*t*	HP (%)
Species composition	NMDS axis 1	0.47	2.92**	97.9	-0.36		0.05		0.93	
	NMDS axis 2	0.22	0.52		-0.19		3.43**	93.5	-0.48	
Guild composition	NMDS axis 1	0.28	2.89**	60.9	-0.18		2.09		1.11	
	NMDS axis 2	0.22	1.57		-1.83		0.28		-1.28	
Assemblage parameters	Number of species	0.53	0.50		-2.09*	33.1	-2.98*	36.2	-1.37	
	Relative abundance	0.25	-2.71*	57.3	-0.51		-1.88		-1.22	
Guild abundance	Frugivores	0.22	-2.35*	38.0	0.01		-2.39*	49.1	-1.26	
	Animalivores	0.33	1.88		-2.12*	32.5	0.76		-1.57	
	Nectarivores	0.67	-3.89***	68.0	-2.52*	11.7	1.16		-2.12*	20.5
	Omnivores	0.10	-0.93		-1.73		-0.48		-1.33	
	Sanguinivores	0.14	1.38		-1.50		-0.04		1.72	

*P < 0.01.

**P < 0.001.

***P < 0.0001.

### Effects of the formation of the dam in the composition of species and guilds of bats

**Fig 4:**
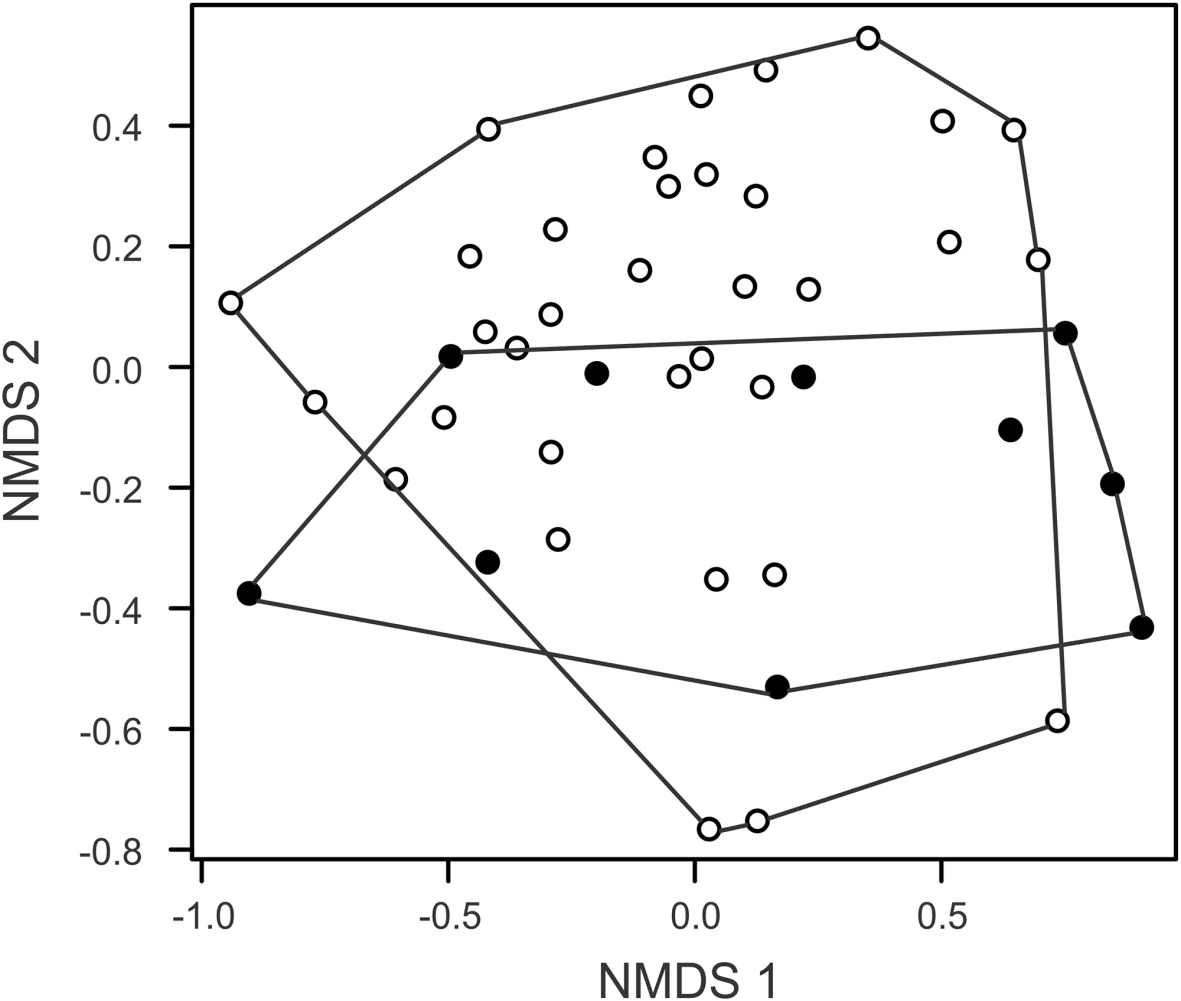
Non-metric multidimensional scaling ordination (NMDS) of the bat species composition between sampling plots that were planned to be submerged by the dam (black circles) and those that will remain dry (open circles) at the Santo Antônio hydroelectric power station area, Southwestern Amazonia, Brazil.

Our level of completeness for the inventories reached 76.2% (Jackknife 1 = 62.6 ± 4.3) for the submerged plots, and 76.1% (Jackknife 1 = 66.9 ± 3.1) for the dry plots (S3 Appendix). There were no differences in the number of species (*t* = 1.08, *P* = 0.30) and capture rates of phyllostomid bats (*t* = 0.35, *P* = 0.73) between the plots remaining dry and the plots going to be submerged. The rarefaction curves of the species richness presented great overlap, reinforcing the similarity of the number of species between the areas (S3 Appendix). On the other hand, the composition of phyllostomids (Fig 4) differed between plots remaining dry and plots to be submerged (ANOSIM, Global R = 0.22, *P* = 0.02) with a more obvious separation along axis 2. The ordination of the plots along the two NMDS axes was responsible for 80.3% of the variation in the species composition (Stress = 0.17) indicating an adequate representation of the data. The two sets of plots shared 72.9% of the total of the species of phyllostomids (35 of the 48 species). Four species were captured only in the plots going to be submerged and nine species were restricted to the dry plots (Table 1). Furthermore, 20 phyllostomid species (41.7%), most of them rarely captured (≤ 10 captures) had at least one third of captures in plots that going to be submerged. The relative abundance of the most widespread species (*A*. *lituratus*, *Lonchophylla thomasi*, and *R*. *pumilio*) were larger in the plots to be submerged (*P* < 0.05).

**Fig 5:**
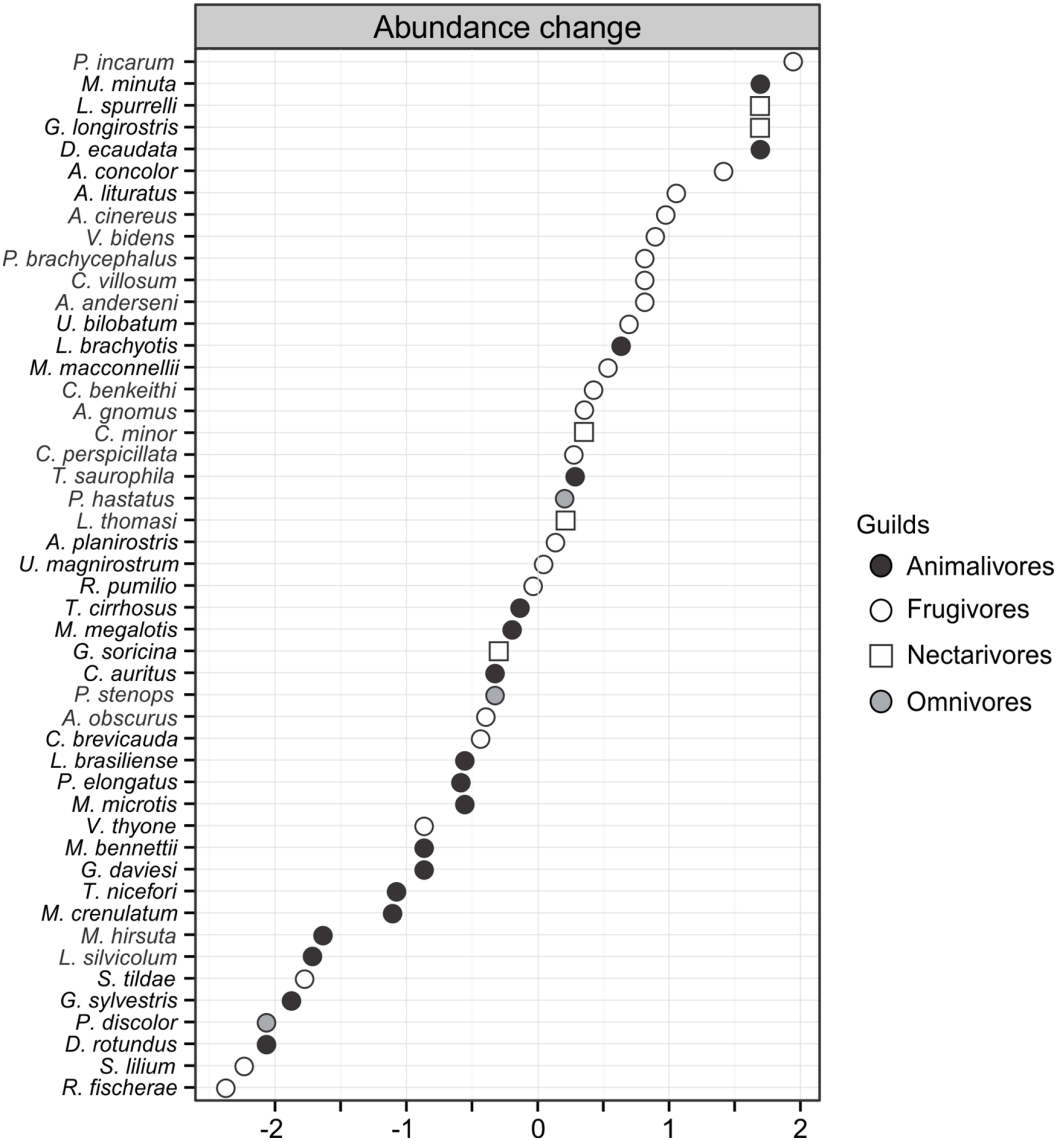
Expected changes in abundance of 48 bat species due to flooding of the Santo Antônio hydroelectric dam, Rondônia State, Brazil, quantified as abundance changes. Changes of abundance greater than zero indicate species with more captures in plots that going to be submerged by the dam. Black circles: animalivores; white circles: frugivores; grey circles: omnivores; white squares: nectarivores.

Overall, frugivores had more species (22) and individuals (1794 bats), followed by animalivores (16 species, 325 bats; Table 2). The ordination of the plots along the two NMDS axes was able to capture 97.8% of the guild composition variation (Stress = 0.06). Guild composition was not different between the sets of plots going to be submerged by the dam and those remaining dry (ANOSIM, Global R = 0.011, *P* = 0.08). Animalivores however (*t* = 2.47, *p* = 0.024) were more abundant in the plots remaining dry (Fig 5). In contrast, there were more frugivores (*t* = 3.01, *P* = 0.007) in the plots going to be submerged (Fig 5).

**Table 2:** Species richness (S), number of captures (N), and relative abundance (%) of bat guilds at Santo Antônio hydroelectric dam area, Southwestern Amazonia, Brazil.

Guilds	Plot											
	Submerse				Dry				Total			
	S	N	%	Mean±SD	S	N	%	Mean±SD	S	N	%	Mean±SD
Frugivore	19	504	82.5	50.4±37.9	22	1290	76.1	37.9±20.3	22	1794	77.8	40.8±25.4
Gleaning animalivore	12	46	7.5	4.6±3.9	15	279	16.5	8.2±4.6	16	325	14.1	7.4±4.6
Nectarivore	5	23	3.8	2.3±1.4	3	58	3.4	1.7±1.7	5	81	3.5	1.8±1.7
Aerial insectivore	9	33	5.4	3.3±3.4	7	44	2.6	2.8±3.5	10	77	3.3	1.8±2.7
Omnivore	2	4	0.7	0.4±0.7	3	19	1.1	0.6±1.0	3	23	1.0	0.5±1.0
Sanguinivore	1	1	0.2	0.1±0.3	1	5	0.3	0.2±0.4	2	6	0.3	0.1±0.3
Total	48	611	100.0	69.5±23.0	51	1695	100.0	47.4±25.9	58	2306	100.0	52.4±26.7

## Discussion

Data collected prior to the establishment of hydroelectric power stations have rarely been made available to serve as baseline for monitoring programs. Because we began our sampling of the Santo Antônio areas before any deleterious effects from upcoming impacts of the dam were incorporated in the system, we were able to make predictions on to what extent the bat fauna and associated habitats could be affected in the upper Madeira river area. The species composition of the phyllostomid assemblages at upper Madeira was different between plots that were planned to be submerged and those planned to remain dry. Also the distribution of bat guild compositions was heterogeneous, and correlated primarily with vegetation-related environmental factors.

Making predictions of the impacts caused by the implementation of a large hydroelectric power station is a multifaceted task, because each species has a different suite of ecological requirements and evolutionary constraints [49]. Differences in species composition were also observed for lizard and bird assemblages studied in the same plots [50] before dam construction suggesting a possible concurrent response of different animal communities to landscape changes caused by hydroelectric dams, on a regional scale.

Bat communities appear to take a long time to recover from the impacts caused by hydroelectric power station, as in the case of the more than 100 years-old areas surrounding the Lake Gatun of Panamá, which appear to have low rates of species substitutions, and somewhat stabilized bat assemblages [11]. Changes in the environment may also not cause immediate measurable changes in communities. Some evidence points to the occurrence of time lags of up to three-years between a given impact and the observation of unstable species richness and/or unstable compositions of bat assemblages [27]. However our results indicated possible severe and faster effects of the UHE Santo Antônio dam because of the combination of unavoidable changes in the flooded areas (as in the permanent flooding of *várzea* forests), and of the singularity of the landscape of each area and their associated animal assemblages that may not be replaceable. These effects can be still more intense under natural disaster scenarios when additive effects to the dam can be expected as in the case of the large flooding of the River Madeira that took place in 2014, partially caused by the River Beni flood in Bolivia. Headwater originated floods in the regions nearby Amazonian rivers have been becoming more intense and frequent in the last years, and that have been caused both by the high temperatures recorded in the equatorial Pacific, and phenomena such as La Niña, which tends to increase rainfall. Water level management are unlikely to mitigate large flood effects, and the opening of dam gates will probably flood the municipality of Porto Velho, which is located downstream of the dam.

The Madeira River carries large amounts of nutrient-rich sediments annually deposited in the riverbanks during its annual flooding [31]. Those fertile soils may support higher abundance and biomass of several species including primates [51,52], and bats [16,53], because of the greater production of fruits. Overall, the areas with the lowest elevations within our sampling plots had higher species richness than those at higher elevations, and had greater abundance of bats, particularly of frugivores. Thus, the permanent flooding of the River Madeira riverbanks can damage rich areas with multiple ecological gradients associated to lower elevations, such as in the case of the areas of *várzea* [16]. Frugivorous and animalivorous bats increase their abundance during high-water season in the Amazonian *várzea* forests [16]. The river level will be changed by the implementation of dam, and the naturally predictable and seasonal flooding pulse in the region will be extinguished. In addition, the high areas that may be eventually flooded are not real *várzea* forests, since the *várzea* forest was suppressed by the UHE. Species associated with the flood pulse and *várzea* forests must adapt to the new flood regime imposed by the dam, and that should be measured.

Several studies have indicated that foliage-gleaning bats are sensitive to environmental disturbance, because of their specialized foraging habits that require a more complex environment, and their highly selective avoidance of forest edges and altered areas [11,54]. However, since animalivores were more abundant in the areas not expected to be submerged by the dam, it is possible that their populations will be less vulnerable to flooding. On the other hand, differences in the environmental characteristics of the two sets of plots may trigger changes to the richness and to the abundance of frugivores. The topographic variables (elevation and slope) varied between the two sets of plots, and flooding of the areas situated in lower elevations imposes losses to the vegetation gradients required for the maintenance of fruit-eating bats.

Landscape variation of the vegetation and of the topography has been shown to be fundamental in shaping the composition of bat assemblages [14,16,25,55,56]. Topographical variables relevant to our study influenced both the distribution and the composition of bat assemblages. Notably large altitudinal variation correlates with equally large environmental changes, and consequently with the shifting of the composition and structure of bat assemblages [25,55]. In the UHE Santo Antônio area, although elevation gradients were relatively subtle (< 100 m), they were enough to alter the composition of the bat assemblages.

Variations of the elevation of terrains have been linked with gradients of humidity and of soil type (18,20,32), and plants may show different degrees of tolerance to the physicochemical properties associated to these gradients [21]. Low-lying areas generally retain more humidity than do adjacent higher altitude sites, because they are generally associated to watercourses and/or to shallow water tables [20,21]. Thus, even subtle variations of the elevation may influence the composition and distribution of plant species, also interfering in the shaping of the local vegetation structure [57], food availability, air humidity, and ultimately constraining directly the habitat use by the understory bats. Recent studies conducted in the Central Amazonia have further demonstrated that the elevation has strong relations with the abundance and mass of fruits and insects consumed by bats [58, Capaverde Jr., personal communication]. Vegetation tends to be more open in lower areas, allowing to easier circulation of bats, in particular of frugivorous species. Moreover, some frugivores remaining closer to areas that are more humid near the riverbank and the *várzea* forests.

Our results also allowed us to record responses in relation to the variations of the vegetation structure from the perspective of guilds, as the relative abundance of the frugivores, nectarivores, and animalivores responded negatively in relation to the vegetation structure, as has previously been documented elsewhere [14,16,59,60]. Habitat use by bats is directly affected by the physical obstructions present in the forested habitats [14]. Bats try to avoid navigating in extensive cluttered vegetation because it hinders their flight and the reception of echolocation signals used to detect the potential obstacles and prey [61–63]. The abundance of frugivores and nectarivores in the UHE Santo Antônio area was associated to the forest basal area that account for the occurrence of large obstacles in the vegetation. In contrast, the relative abundance of animalivores was correlated with a finer component of the vegetation, which was the arrangement of leaves and branches.

The responses of the phyllostomid guilds to diverse vegetation characteristics have ultimately been related to wing morphology, foraging mode, and echolocation behavior [64,65]. Frugivores and nectarivores are adapted to search for widely dispersed resources since they consume fruits and nectar from patchily distributed shrubs and trees, but they are constrained by their higher wing loading and lower aspect ratios [64–66]. Trunks may represent major obstacles that limit movements between feeding areas, and dense vegetation will require greater maneuverability, which is very expensive energetically, and even more if flights are to be longer. In contrast, animalivores tend to forage over lower distances in a daily basis when searching for prey, and to rely in acoustic orientation by listening to the sounds generated by the arthropods on the surrounding vegetation and soil [61,62]. In these cases, echolocation calls may provide to the bats most of the necessary cues to locate and capture their prey. Short and broad wings with rounded tips allow to a finely maneuverable and slow flight facilitating the capture of prey in the substrate [64–66]. However, even the specialized abilities of the animalivores to capture prey may be limited by the position of the prey on the vegetation. Smaller obstacles generated by the intricate architecture formed by the arrangement of the leaves and the branches in the vegetation can obstruct the reception of the echolocation calls of animalivores, and decrease the efficiency to detect, classify, locate, and to catch their preys.

The plan of energetic expansion 2017–2021 from the Ministry of Mines and Energy of Brazil, which include the construction of nine large hydroelectric dams in the Brazilian Amazon, show an urgent call to adjust the energetic needs of the country to a scenario of lower impacts in the Amazonian natural landscapes. These large buildings need to be accompanied by robust biodiversity monitoring plans conducted pre and post landscape impact and in pristine and altered environments [67]. In order to be useful, studies of the impact caused by the implementation of large hydroelectric power stations must be conducted considering different levels of organization (composition, richness and abundance of species and guilds), and to test how local conditions influence and shape biodiversity. It is of paramount importance that such studies are conducted prior to the initiation of any disturbances associated with the establishment of the hydroelectric stations, departing from the zero from the whole communities’ perspective, and so increase the chances of their resilience and future long-term survival. At the moment, we do not know the resilience of the bat species, how they will respond to vegetation changes and the new regime of the flood pulse imposed by dam, and we know nothing about their ability to migrate to drier forested areas. However, we can predict that large environmental changes will bring deleterious effects to local biodiversity and that proactive management strategies may improve chances of survival for several species, including bats. As management strategies, we suggest the expansion and/or creation of conservation units in the directly affected area of the dam, with special attention to lowlands areas and to resources considered as conservation hotspots for the bats of the Madeira river, such as the riverbed rocky outcrops [48]. The Mapinguari National Park, with 1,776,914.18 ha, is a conservation unit in the region that may perhaps play a role in the mitigation of the impacts of the dam. Another point is to guarantee logistical and financial assistance for long-term biodiversity monitoring in the region.

## Supporting information

S1 Appendix Summary of sampling effort, number sampling nights, and dam effect in in the 44 sampling plots that will be submerged and those that remain dry by the Santo Antônio hydroelectric dam, Western Amazonia, Brazil. (XLSX)

S2 Appendix List of bat species, guilds, occurrence per sampling plot, and sampling effort of captures in the 44 sampling plots that will be submerged and those that remain dry by the Santo Antônio hydroelectric dam, Western Amazonia, Brazil. (XLSX)

S3 Appendix Species-accumulation curves for phyllostomid bats captured in the 44 sampling plots that will be submerged and those that remain dry by the Santo Antônio hydroelectric dam, Western Amazonia, Brazil. Dashed lines represent 95% confidence intervals, grey line and circle represent flooded plots, and dark line and circle represent dry plots. Circles indicates the estimated number of species (± SD) based on the Jackknife 1 estimator.
(TIF)
